# Inhibitory and facilitatory effects of phonological and orthographic similarity on L2 word recognition across modalities in bilinguals

**DOI:** 10.1038/s41598-021-92259-z

**Published:** 2021-06-17

**Authors:** Candice Frances, Eugenia Navarra-Barindelli, Clara D. Martin

**Affiliations:** 1 BCBL, Basque Center On Brain, Language and Cognition, Donostia, Spain; 2 Department of Social Sciences and Law, UPV/EHU, Donostia, Spain; 3 Basque Foundation for Science, Bilbao, Spain

**Keywords:** Language, Human behaviour, Cognitive neuroscience

## Abstract

Language perception studies on bilinguals often show that words that share form and meaning across languages (cognates) are easier to process than words that share only meaning. This facilitatory phenomenon is known as the cognate effect. Most previous studies have shown this effect visually, whereas the auditory modality as well as the interplay between type of similarity and modality remain largely unexplored. In this study, highly proficient late Spanish–English bilinguals carried out a lexical decision task in their second language, both visually and auditorily. Words had high or low phonological and orthographic similarity, fully crossed. We also included orthographically identical words (perfect cognates). Our results suggest that similarity in the same modality (i.e., orthographic similarity in the visual modality and phonological similarity in the auditory modality) leads to improved signal detection, whereas similarity across modalities hinders it. We provide support for the idea that perfect cognates are a special category within cognates. Results suggest a need for a conceptual and practical separation between types of similarity in cognate studies. The theoretical implication is that the representations of items are active in both modalities of the non-target language during language processing, which needs to be incorporated to our current processing models.

## Introduction

At this point in the literature, it is quite clear that the two languages of a bilingual influence each other—meaning that a bilingual is not simply two monolinguals in one body^1^. This idea is highlighted by the concept of language co-activation, which suggests that neither language of a bilingual is ever fully inactive or “off”. The present study explores this idea by assessing whether second language (L2) word recognition—both visual and auditory—is influenced by the phonological and orthographic similarity between translation equivalents.

When exploring the existence of co-activation—namely, the activation of the non-target language on target-language processing—, one of the most frequent evidences is the cognate effect. Cognates are defined as translations that share both form and meaning across languages (e.g., ‘paper’ and ‘papel’, in English and Spanish, respectively) as opposed to non-cognates, which share only meaning across languages (e.g., ‘book’ and ‘libro’). The cognate effect refers to the idea that words that are similar in form between languages often produce facilitatory effects on word recognition in a unilingual context^2–9^. Note that there is also a particular case of cognates in which words are not simply spelled similarly between languages but rather are exact matches, or orthographically identical words (identical cognates). These perfect or identical cognates are thought to have a special status, causing larger effects than non-identical cognates^10^.

Despite a large amount of literature exploring the cognate effect, the focus has been quite narrow, placing the emphasis on orthographic similarity and using visual word recognition paradigms^11–15^. Orthographic similarity manipulations in the visual modality consistently lead to a facilitation effect of cognates in perception. This cognate advantage—or cognate facilitation effect—is interpreted as a co-activation of both languages (the target and the non-target). As cognates share form and meaning across languages, the associated activation between those words is larger (higher resting level activation) compared to non-cognates (lower resting level activation), explaining their higher processing speed^5,10,16,17^.

Although the orthographic similarity effect in the visual modality is well documented, phonological similarity has received little attention and very few studies have explored the cognate effect aurally. Within the few studies exploring the cognate effect aurally, most have determined the cognate status of words based on orthographic similarity, and the results are not conclusive. Some studies have found that cognates are not always facilitatory and can even be inhibitory. In particular, one study found that L2 proficiency modulates the effect of orthographic similarity orally, with higher proficiency participants showing facilitation and lower proficiency participants showing inhibition with increased similarity^18^. Thus, we know that there is a facilitatory cognate effect in the visual modality when manipulating orthographic similarity, but there is a need to explore the cognate effect in the auditory modality when manipulating phonological similarity independently from orthography. This is one of the main objectives of this study.

Within the set of studies testing the cognate effect taking into account phonological similarity, most have focused on production, particularly using picture naming tasks. Sadat and colleagues^19^ found a phonological facilitatory cognate effect in naming latencies using ALINE distance to measure phonological similarity. They observed that pictures of cognate words were produced faster than those of non-cognates. Schwartz, Kroll, and Diaz^8^ manipulated phonological and orthographic similarity orthogonally between cognates and perfect cognates (orthographically identical words sharing meaning) in a picture naming task. They found that with high orthographic similarity, naming latencies were slowed by dissimilar phonology. It should be noted that they compared only cognates and perfect cognates, thus their results are not easily generalizable to all vocabulary. In one of the few studies exploring phonological similarity effects in perception, Dijkstra and colleagues^5^ assessed native Dutch speakers with a high level of English proficiency on a visual lexical decision task (LDT). They manipulated phonological and orthographic similarity between Dutch and English and found a facilitation in LDT performance—in both response time and percent errors—for words that shared either only form—namely, false friends—or both form and meaning—i.e., cognates—across languages. On the other hand, recognition latencies were delayed when words only shared their phonology—and not orthography—across languages. Although they manipulated phonology and orthography orthogonally, they only provided pairwise comparisons and no information on interactions between the two types of similarity. Therefore, based on few prior studies, phonological similarity might not be as consistently facilitatory as orthographic similarity is, and some results suggest that phonological and orthographic similarity might even interact. This shows a need for further studies exploring both phonological similarity and the relationship between orthographic and phonological similarity in both the visual and auditory modalities.

A further issue of working in the auditory modality is establishing measures of similarity. Within those studies assessing the cognate effect aurally, measurements of similarity have varied widely in their level of objectivity and often centered on orthographic similarity as mentioned above. For one, Grasso and colleagues^20^ considered items that were phonological cognates or non-cognates based on the subjective assessments of three bilingual pathologists. Schwartz and Kroll^21^ used a combination of a subjective assessment by the experimenters, two bilinguals, and monolingual English speakers, with an objective assessment based on orthographic similarity^14^. Others have used more objective measures using what is called string alignment. These measures quantify the number of operations (insertions, deletions, and substitutions) necessary to convert one string—e.g., graphemes or phonemes—into the other. Some authors have proposed using Levenshtein distance, which uses alignment of graphemes^22,23^, while Sadat and colleagues^19^ used ALINE distance, which instead aligns phoneme strings, taking into account the features of the phonemes it compares^24^.

The Levenshtein distance formula mentioned before is the most common algorithm used to define orthographic similarity, but unfortunately, it approximates phonological similarity from orthographic similarity. If the two languages that are compared share grapheme to phoneme mappings—or, at least, they have mostly one-to-one correspondences between phonemes and graphemes—then this would not be a major concern as orthographic and phonological similarity would align well. Nevertheless, this is often not the case, as many languages do not have direct, one-to-one mappings between orthography and phonology, as is the case of English with, e.g., “write”, “right”, and “rite” all pronounced the same. In addition, even if the languages have direct mappings between phonemes and graphemes, these may differ between languages—e.g., “piano” in Spanish and “πιάνο” in Greek or “sh” in English and “sch” in German where each pair is pronounced the same, but spelled differently. Finally, approximating phonological similarity from orthographic similarity is also a concern for cases in which languages share the orthographic system but the phonological repertoire is quite different. This is the case between Spanish and English, in which “violin” (spelled the same in both languages) is pronounced /bio'lin/ and /'vaɪəlɪn/, respectively. Here, the similarity measured using orthographic Levenshtein distance would say those words are identical, but in fact, in the auditory modality, they only share two phonemes. Using orthographic similarity in auditory word recognition might then not be a good approximation. This is particularly the case in the commonly used language pair, English and Spanish.

Importantly, the cases above in which orthographic and phonological similarity do not align might carry with them an interaction between these degrees of similarity which further complicates the picture. This suggests a need not only for exploring the effect of phonological similarity, but also its possible interaction with orthographic similarity, both in the visual modality and the understudied auditory modality. This will be our second aim.

## The present study

In order to achieve the aforementioned goals, we ideally needed a pair of languages which follow different phoneme-to-grapheme conversion rules, allowing us to manipulate orthographic and phonological similarity orthogonally. Although orthography and phonology tend to correspond to one another, English, with its opaque orthography—i.e., one-to-many and many-to-one phoneme-to-grapheme correspondences—, allows for the two to be dissociated. Furthermore, English and Spanish share the same alphabet but have many differences in the number and identity of phonemes. Thus, we tested Spanish–English bilinguals using English as the target language. We tested this group of participants on lexical decision tasks in two modalities—visual and auditory—in order to compare participants’ performance on the same items across modalities.

Cognates that are orthographically—although not necessarily phonologically—strictly identical between languages (e.g., “violin”, /'vaɪəlɪn/ in English and /bio'lin/ in Spanish) will be referred to in the current text as orthographically identical words to highlight phonological differences in our language pair. As mentioned before, some researchers claim that translations that are orthographically identical between languages (often called perfect cognates) have a special status^3,10^, whereas others consider them part of the spectrum of orthographic similarity^8,11^. As a secondary goal, we have included orthographically identical words to test whether they are categorically different from non-identical cognates. Note that we focused only on identical orthographic cognates and not identical phonological cognates, given that the latter group is extremely rare in the Spanish–English language combination.

We used ALINE distance as objective measure of phonological similarity, as it has been shown to be better than those used in prior studies^25–29^. Part of its advantage is that it takes into consideration features of the phonemes, and thus provides a more precise comparison for phonological strings than, for example, Levenshtein distance. ALINE also aligns strings rather than just comparing them, which allows for a better comparison when differences between words in the two languages imply the insertion or deletion of several phonemes or graphemes^29^.

In summary, the aims of the present study were to (1) test the effects of phonological similarity on auditory word recognition, (2) assess the influence of phonological and orthographic similarity between modalities—visual and auditory—, and (3) evaluate whether orthographically identical words have a special status.

## Method

### Participants

Participants were 55 native Spanish speakers (F = 34, M_age_ = 26.25 [SD = 6.01]) from Madrid and Murcia, with at least an intermediate level in English (their second language). Participants had a minimum score of 40 on the English BEST^30^—a picture naming task with a maximum score of 65—and of 60% on the English LexTALE (a vocabulary test), which equates to approximately a B2 (upper intermediate) level^31^. Participants’ average score on the BEST was 57.58 (SD = 5.86) with a range of 40 to 64. With respect to the LexTALE, their average score was 77% (SD = 10%) with a range of 60 to 99%. Participants self-reported their average daily exposure of English, which was on average 34% of the time (SD = 22%). Their average self-reported age of acquisition of English was 6.5 (SD = 2.5) years old, with a minimum of 3 years of age. All participants provided informed consent before taking part in the experiment, which was conducted in accordance with the Declaration of Helsinki and approved by the Basque Center on Cognition, Brain and Language ethics committee (approval number 9994). Participants were paid for taking part in the experiment.

### Stimuli

**Figure 1:**
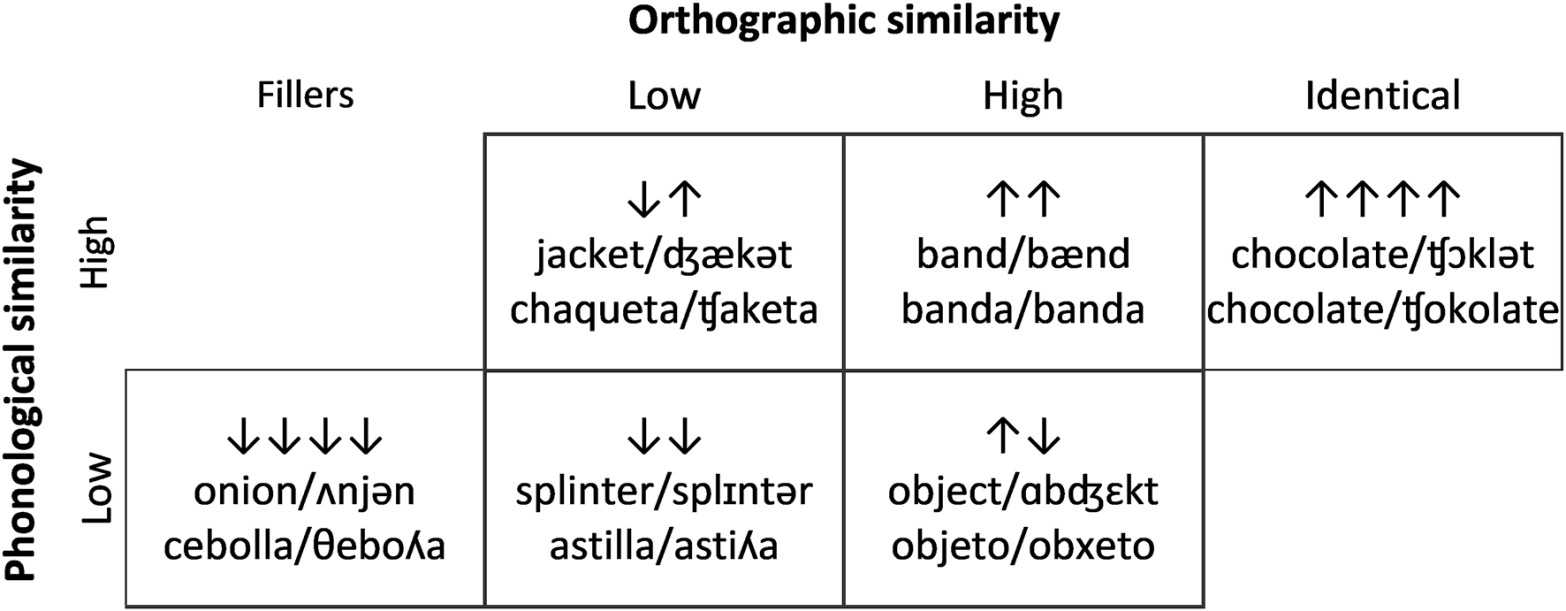
Orthographic and phonological similarity distribution and examples of words in each category. The reported phonological transcriptions correspond to a general American English accent, which is the accent of the speaker who recorded the stimuli. Pseudowords were recorded to match the IPA transcription rather than the orthography of the pseudoword. All transcriptions were verified by a linguist to match the stimuli. Each row represents a level of phonological similarity and each column represents a level of orthographic similarity. Each cell contains 50 items.

Stimuli consisted of 300 words and 300 pseudowords. Words were divided into six categories. Four of these consisted of a Latin square between orthographic and phonological similarity. Another one contained items in the extreme of the orthographic similarity distribution—orthographically identical words—and the last group consisted of extreme dissimilarity. The latter group was included in order to balance the number of high and low similarity items, and is considered hereafter a group of fillers (see Fig. 1 for an example of each). For the main manipulation, we had four groups: high phonological and orthographic similarity (P_High_O_High_), high phonological similarity and low orthographic similarity (P_High_O_Low_), low phonological similarity and high orthographic similarity (P_Low_O_High_), and low phonological and orthographic similarity (P_Low_O_Low_). Apart from those 4 main groups, there was the orthographically identical translation group (P_High_O_Iden_), which was included to assess the “special status” of those items, on each of the modalities. Note that no identical phonology (or phonologically identical) group was included, since the differences in phonology between languages made it impossible to find enough items. High and low phonological and orthographic similarity were defined by median split. For phonology, we used inverse Aline distance, with the median split at 0.74. The high similarity range of inverse Aline distance values was 0.741 to 0.951 and for the low similarity group, the range was 0.195 to 0.736. For orthography, we used the same measure (inverse Aline distance) with the median split at 0.77. The high similarity range of inverse Aline distance value was 0.771 to 0.982 and for the low similarity group, the range was 0.360 to 0.769.

All six groups of items were matched on the following variables: word frequency (raw and logarithmic), frequency of the Spanish translation (raw and logarithmic), number of syllables, number of letters, and number of phonemes (see Table 1 for means, standard deviations, and statistics), all extracted from CLEARPOND^32^. Pseudowords were created by exchanging the last two phonemes (2 or 3 letters) between words used in the task (e.g., lens/lɛnz changed to lert/lɛrt and airport/ɛrpɔrt to airpons/ɛrpɔnz). This way, the number of letters and phonemes remained constant and all items had to be listened up to the penultimate phoneme in order to differentiate the word from the pseudoword. In other words, we maintained the uniqueness point of target words constant between stimuli and as late as possible. Furthermore, we utilized an orthographic approximation of these words for the visual stimuli to maintain the two modality conditions as similar as possible. Finally, this way of creating stimuli allowed us to match the pseudowords to each of the target word categories and thus calculate A’ for each group to assess performance.

**Table 1:** Means, standard deviations, and statistics for variables stimuli were matched on.

Orthographic similarity	Low		High		Identical	
Phonological similarity	Low	High	Low	High	High	Statistic
English frequency	25.21 (21.85)	30.81 (43.55)	42.61 (80.29)	24.63 (21.65)	31.64 (49.27)	*F*(4,245) = 1.117, *p* = .349, *BF*_01_ = 16.175
English log frequency	1.22 (0.45)	1.12 (0.61)	1.27 (0.57)	1.23 (0.40)	1.17 (0.51)	*F*(4,245) = .667, *p* = .616, *BF*_01_ = 32.943
Spanish frequency	50.37 (56.59)	67.63 (80.91)	79.34 (106.46)	58.89 (58.58)	69.22 (101.77)	*F*(4,245) = .864, *p* = .486, *BF*_01_ = 24.147
Spanish log frequency	1.38 (0.63)	1.47 (0.65)	1.47 (0.69)	1.48 (0.64)	1.38 (0.72)	*F*(4,245) = .304, *p* = .875, *BF*_01_ = 58.305
Number of syllables	2.00 (0.88)	2.08 (0.92)	2.00 (0.90)	1.86 (0.76)	1.88 (0.39)	*F*(4,245) = .670, *p* = .613, *BF*_01_ = 32.769
Number of letters	6.36 (1.96)	6.08 (2.06)	6.52 (1.76)	6.38 (2.00)	5.84 (1.17)	*F*(4,245) = 1.126, *p* = .345, *BF*_01_ = 15.940
Number of phonemes	5.94 (2.08)	5.98 (2.20)	5.46 (1.76)	5.88 (1.83)	5.56 (1.07)	*F*(4,245) = .839, *p* = .502, *BF*_01_ = 25.110

Values are means with standard deviations in parentheses. The N in all cases is 50.

There was a total of 50 words per condition, for a grand total of 300 words. There were also 50 pseudowords per condition—one matched to each word. All words and pseudowords were presented once in each modality.

Auditory stimuli were recorded in a quiet recording room by a native speaker of English with a general American accent^33^ and following the pronunciation reported in the Carnegie Mellon CMU dictionary^34^. They were normalized to 1 dB and cut with 500 ms of silence before and after, using Audacity^35^. They were recorded at a frequency of 44.1 kHz and 32 bits.

### Procedure

The main task was a lexical decision task. Participants saw a fixation cross for 500 ms and then either heard the phoneme sequence or saw the letter string and had to decide whether it was a real word. The words were visually displayed on a 17″ screen with a definition of 1024 by 768 pixels, with a sans-serif font of size 70 px (white text on a black background). The auditory stimuli were presented using headphones playing them in 16-bit WAV format at a 44.1 kHz sample rate. Participants had 2500 ms to respond to the auditory stimuli (starting from the onset of the word) and 1500 ms to respond to the visual stimuli. They responded using the F (real word) and J (pseudoword) keyboard keys. Participants had six practice trials with feedback before moving on to the main task (without feedback). They did the experiment in two sessions one week to 10 days apart. In each session, participants completed either the auditory or the visual task (counterbalanced between participants). The experiment had a duration of 40 min per session with 2 sessions in total. Each session contained 4 blocks with self-paced pauses in between.

### Analyses

The orthographic and phonological similarity effects on the LDT were assessed using two-way analyses of variance (ANOVAs), as well as linear mixed effects models (LMEs) provided in the supplementary materials.

For the ANOVAs, first, we looked at the effects of phonological and orthographic similarity on A’—a sensitivity index that takes into consideration hits and false alarms—using the Psycho package in R^36^. This was carried out once for each modality. A’ was calculated using the accuracy on words to calculate the hit rate and pseudoword errors to calculate the false alarms.

Then, we carried out ANOVAs on the effects of phonological and orthographic similarity on response times. For the auditory modality, we subtracted the duration of the stimulus from the recorded response time—measured from stimulus onset until the response—, to cancel out the effect of the stimulus duration. For response times in general, only correct responses were taken into account and any responses less than 150 ms or more than 2 standard deviations away from the mean in each category for each participant were excluded. For the orthographically identical words, we carried out one-way ANOVAs for the main effect of orthographic similarity, keeping only the high phonological similarity groups. This means that we compared the following three groups: P_High_O_Low_, P_High_O_High_, and P_High_O_Iden_. This was done in order to match the 3 groups on phonological similarity as there were no low phonological similarity items in the orthographically identical group.

Finally, we carried out the same tests by participant and by item and for all tests, we carried out follow-ups using Bonferroni corrections. All *p* values for the post hoc tests are reported with the Bonferroni correction calculated (i.e., *p* value was multiplied by the number of tests). Therefore, the threshold of significance remains 0.05. In all cases, F1 and F2 was calculated, but it should be noted that the by participant analyses were substantially higher powered. This is because F1 was within subjects whereas F2 was between items, with fewer items (50) than participants (55).

Recent work has found that ANOVAs are robust to violations of normality^37,38^. Thus, we have not corrected for violations of the normality assumption.

There was an overall effect of location, such that participants in Madrid performed better than those in Murcia, but the results did not change qualitatively when we included location as a factor. Since it was not a variable of interest, we collapsed the data across locations. All tests were carried out using JASP^39^.

## Results

All participants performed above chance in all conditions (average performance in audio: 77.25% [9.01%] and in visual: 89.33% [6.12%]). Using our criteria, we excluded 5.09% (0.64%) of responses—number of items removed did not vary across conditions, auditory: *F*_*1*_(4, 216) = 1.973, *p* = 0.10, visual: *F*_*1*_(4, 216) = 1.132, *p* = 0.342.

### Auditory task

#### Orthographic and phonological similarity effects

**Figure 2:**
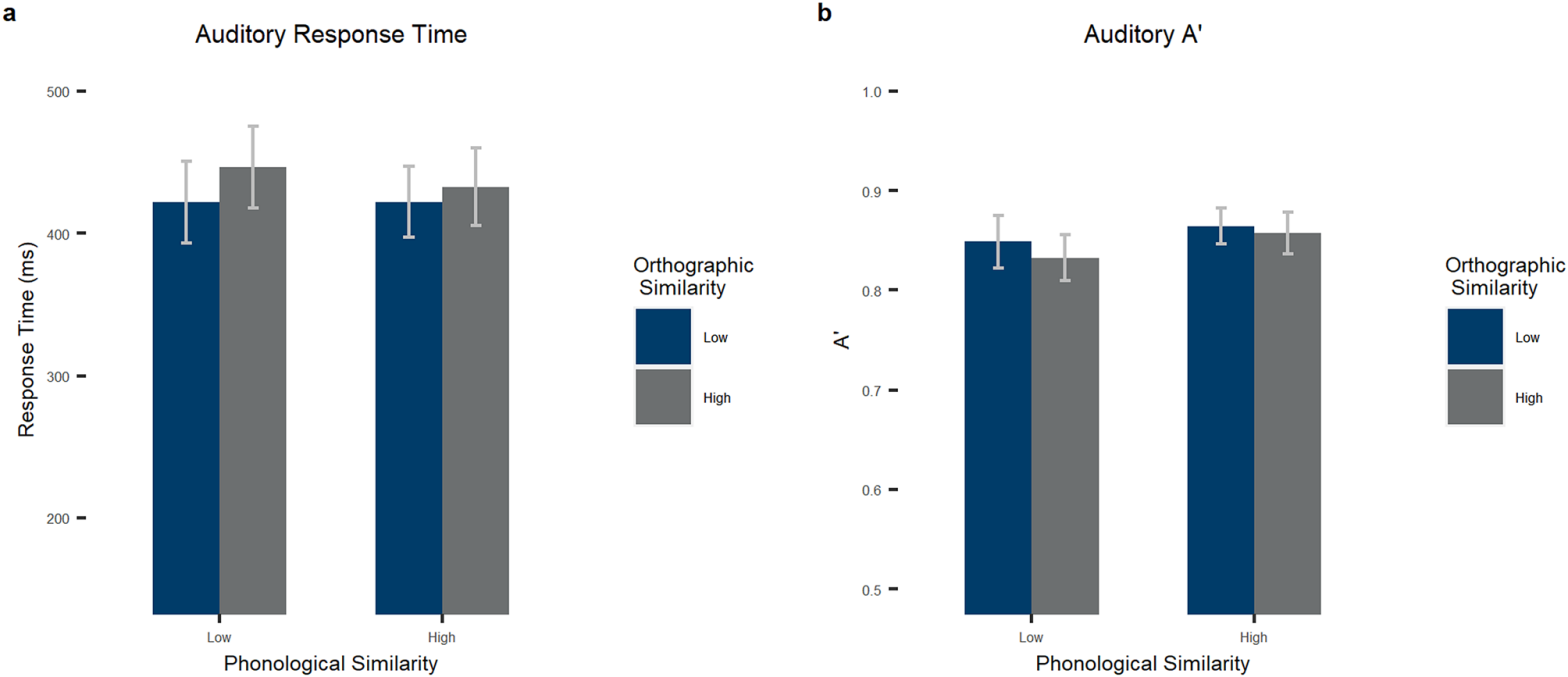
Results for the lexical decision task in the auditory modality. (**a**) Response times in miliseconds and (**b**) A’ by phonological (bar clusters) and orthographic (bar color) similarity for the auditory task. Error bars stand for 95% confidence intervals.

For A’ in the auditory modality, we found a main effect of phonological similarity by participant, such that high phonological similarity led to higher signal detection (M = 0.86, SD = 0.07) than low similarity (M = 0.84, SD = 0.09), *F*_*1*_(1, 54) = 18.349, *p* < 0.001, *η*_*p*_^*2*^ = 0.254; by item, this phonological effect was not significant, *F*_*2*_(1, 196) = 0.478, *p* = 0.490, *η*_*p*_^*2*^ = 0.002. There was also a main effect of orthographic similarity by participants, such that high orthographic similarity led to lower signal detection (M = 0.85, SD = 0.08) than low similarity (M = 0.86, SD = 0.08), *F*_*1*_(1, 54) = 6.211, *p* = 0.016, *η*_*p*_^*2*^ = 0.103 (no effect by item, *F*_*2*_(1, 196) = 1.273, *p* = 0.261, *η*_*p*_^*2*^ = 0.006). Finally, there was no interaction between the two factors, *F*_*1*_(1, 54) = 0.838, *p* = 0.364, *η*_*p*_^*2*^ = 0.015, *F*_*2*_(1, 196) = 0.518, *p* = 0.472, *η*_*p*_^*2*^ = 0.003. See Table 2 and Fig. 2 for by-subject statistics.

**Table 2:** By-subject means and standard deviations.

Orthographic similarity		Low		High		Identical
Phonological similarity		Low	High	Low	High	High
Auditory	A'	0.85 (0.10)	0.86 (0.07)	0.83 (0.09)	0.86 (0.08)	0.86 (0.09)
	RT (ms)	429.78 (108.98)	427.89 (94.80)	452.36 (109.14)	439.32 (103.00)	480.01 (104.12)
Visual	A'	0.94 (0.05)	0.93 (0.05)	0.94 (0.04)	0.94 (0.04)	0.96 (0.03)
	RT (ms)	670.63 (71.87)	683.38 (77.88)	670.63 (71.87)	673.83 (74.23)	626.78 (70.26)

Numbers in parentheses are standard deviations. The N in all cases is 55.

For response time in the auditory modality, we found no main effect of phonological similarity, *F*_*1*_(1, 54) = 1.707, *p* = 0.197, *η*_*p*_^*2*^ = 0.031, *F*_*2*_(1, 296) = 0.721, *p* = 0.396, *η*_*p*_^*2*^ = 0.002. There was a main effect of orthographic similarity, such that high orthographic similarity led to longer reaction times (M = 445.84, SD = 102.99) than low similarity (M = 428.83, SD = 98.29), *F*_*1*_(1, 54) = 12.078, *p* < 0.001, *η*_*p*_^*2*^ = 0.183 (marginally significant by stimulus, *F*_*2*_(1, 196) = 2.840, *p* = 0.094, *η*_*p*_^*2*^ = 0.014). Finally, there was no interaction between the two factors, *F*_*1*_(1, 54) = 1.360, *p* = 0.249, *η*_*p*_^*2*^ = 0.025, *F*_*2*_(1, 196) = 0.558, *p* = 0.456, *η*_*p*_^*2*^ = 0.003. See Table 2 and Fig. 2 for by-subject statistics.

#### Identical orthography items (orthographically identical words)

For A’ in the auditory modality, we found no effects of orthographic similarity in the by-subject analysis, *F*_*1*_(2, 108) = 0.732, *p* = 0.483, *η*_*p*_^*2*^ = 0.013, nor in the by-item analysis, *F*_*2*_(2, 147) = 0.037, *p* = 0.963, *η*_*p*_^*2*^ < 0.001.

**Figure 3:**
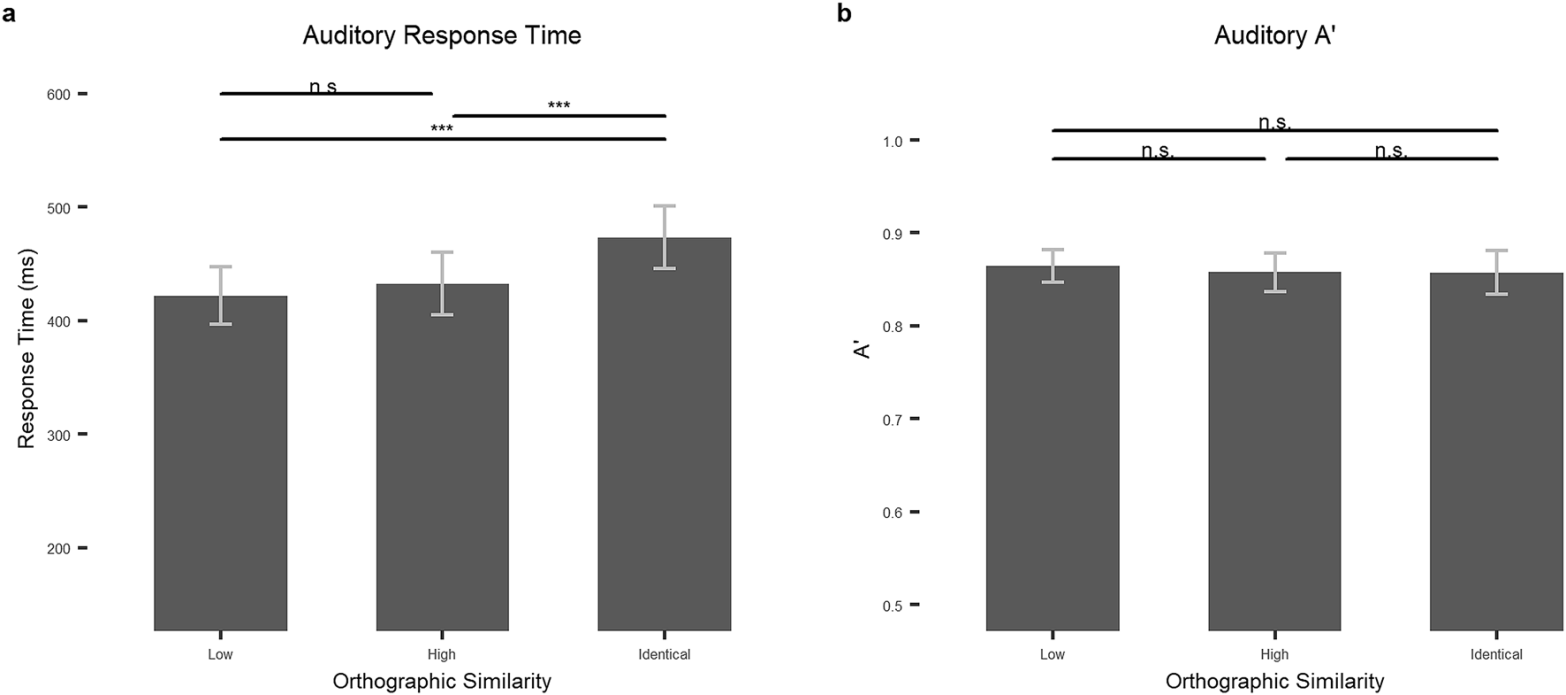
The effects of orthographic similarity on the lexical decision task in the auditory modality. (**a**) Response times in miliseconds and (**b**) A’ by orthographic similarity (low, high, and identical) for the auditory task. Error bars stand for 95% confidence intervals. N.s.: *p* > .1; + : *p* < .1; *: *p* < .05; **: *p* < .01, ***: *p* < .001.

In the response time analysis, we found an effect of orthographic similarity, *F*_*1*_(2, 108) = 25.801, *p* < 0.001, *η*_*p*_^*2*^ = 0.323, *F*_*2*_(2, 147) = 5.6730, *p* = 0.004, *η*_*p*_^*2*^ = 0.072. In the by-subject post-hoc tests, we found that identical items (M = 480.01, SD = 104.12) were responded to slower than both high similarity (M = 439.32, SD = 104.00), *t*(54) = 5.865, *p* < 0.001, Cohen’s D = 0.791, and low similarity items (M = 427.89, SD = 94.80), *t*(54) = 6.102, *p* < 0.001, Cohen’s D = 0.823, but high and low similarity items did not differ, *t*(54) = 1.563, *p* = 0.371, Cohen’s D = 0.211. See Table 2 and Fig. 3 for by-subject means and standard deviations. Similarly, regarding the by-item analysis, in the post-hoc tests, we observed that identical items (M = 506.62, SD = 93.68) were responded to slower than both high (M = 458.89, SD = 103.37), *t*(54) = 2.507, *p* = 0.040, Cohen’s D = 0.484, and low similarity items (M = 445.26, SD = 87.87), *t*(54) = 3.224, *p* = 0.005, Cohen’s D = 0.676, but high and low similarity items did not differ, *t*(54) = -0.716, *p* = 1, Cohen’s D = 0.142.

### Visual task

#### Orthographic and phonological similarity.

**Figure 4:**
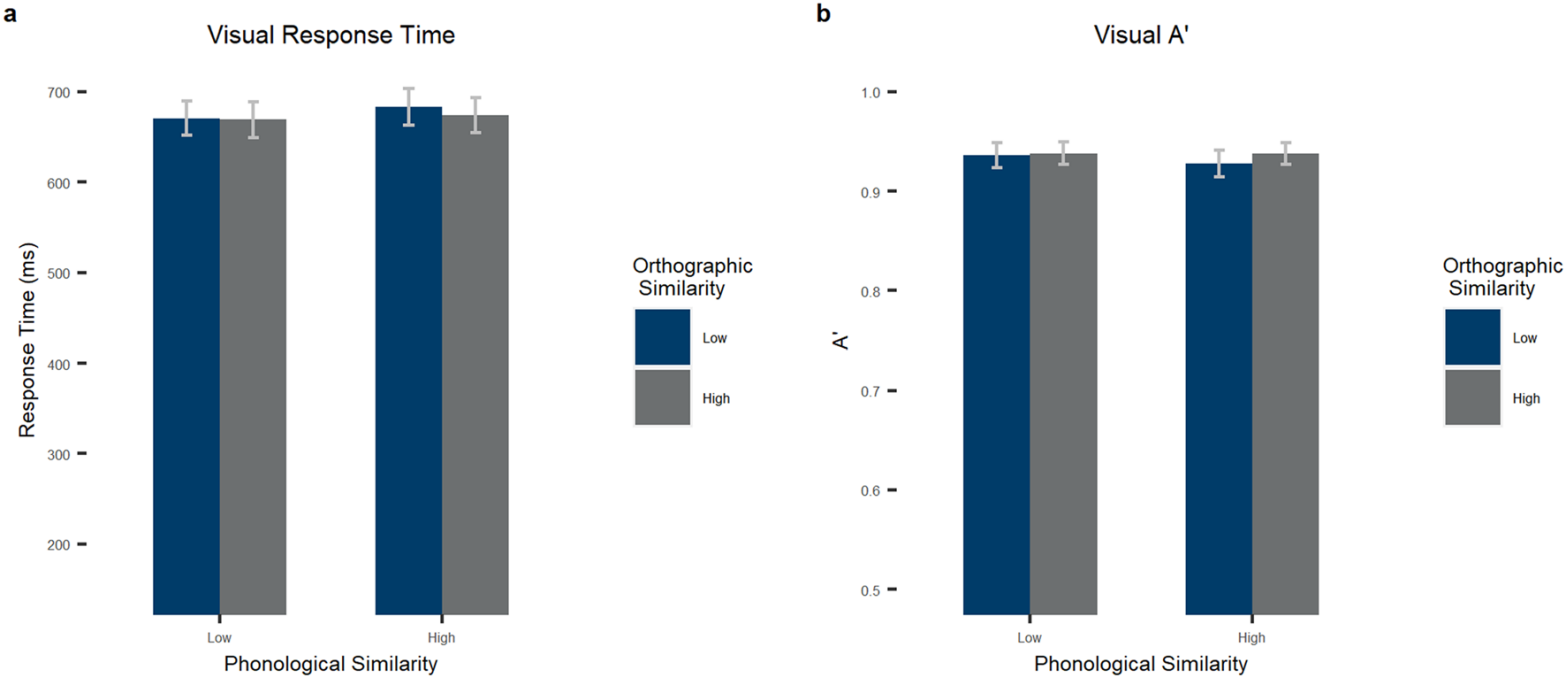
Results for the lexical decision task in the visual modality. (**a**) Response times in miliseconds and (**b**) A’ by phonological (bar clusters) and orthographic (bar color) similarity for the visual task. Error bars stand for 95% confidence intervals.

For A’ in the visual modality, there was a main effect of orthographic similarity, *F*_*1*_(1, 54) = 4.962, *p* = 0.030, *η*_*p*_^*2*^ = 0.084, such that high orthographic similarity led to higher signal detection (M = 0.94, SD = 0.04) than low similarity (M = 0.93, SD = 0.05). In the by-item analysis, we did not find this orthographic similarity effect, *F*_*2*_(1, 196) = 0.434, *p* = 0.511, *η*_*p*_^*2*^ = 0.002. We found a marginal main effect of phonological similarity in the by subject analysis, *F*_*1*_(1, 54) = 2.982, *p* = 0.090, *η*_*p*_^*2*^ = 0.052, such that high phonological similarity tended to led to lower signal detection (M = 0.93, SD = 0.04) than low similarity (M = 0.94, SD = 0.04) (no effect in the by-item analysis, *F*_*2*_(1, 196) = 0.072, *p* = 0.788, *η*_*p*_^*2*^ < 0.001). Finally, there was no interaction between the two factors, *F*_*1*_(1, 54) = 1.896, *p* = 0.174, *η*_*p*_^*2*^ = 0.034, *F*_*2*_(1, 196) = 0.143, *p* = 0.706, *η*_*p*_^*2*^ < 0.001. See Table 2 and Fig. 4 for by-subject statistics.

For response times in the visual modality, there was no main effect of orthographic similarity, *F*_*1*_(1, 54) = 2.319, *p* = 0.134, *η*_*p*_^*2*^ = 0.041, *F*_*2*_(1, 196) = 1.033, *p* = 0.311, *η*_*p*_^*2*^ = 0.005. We found a main effect of phonological similarity in the by-subject analysis, *F*_*1*_(1, 54) = 6.889, *p* = 0.011, *η*_*p*_^*2*^ = 0.113, such that high phonological similarity led to slower response times (M = 678.60, SD = 73.97) than low similarity (M = 669.87, SD = 71.47), (no effect in the by stimulus analysis, *F*_*2*_(1, 196) = 2.484, *p* = 0.117, *η*_*p*_^*2*^ = 0.012). There was also no interaction between the two factors, *F*_*1*_(1, 54) = 1.946, *p* = 0.169, *η*_*p*_^*2*^ = 0.035, *F*_*2*_(1, 196) = 0.902, *p* = 0.343, *η*_*p*_^*2*^ = 0.005. See Table 2 and Fig. 4 for by-subject statistics.

#### Identical orthography items (Orthographically identical words)

**Figure 5:**
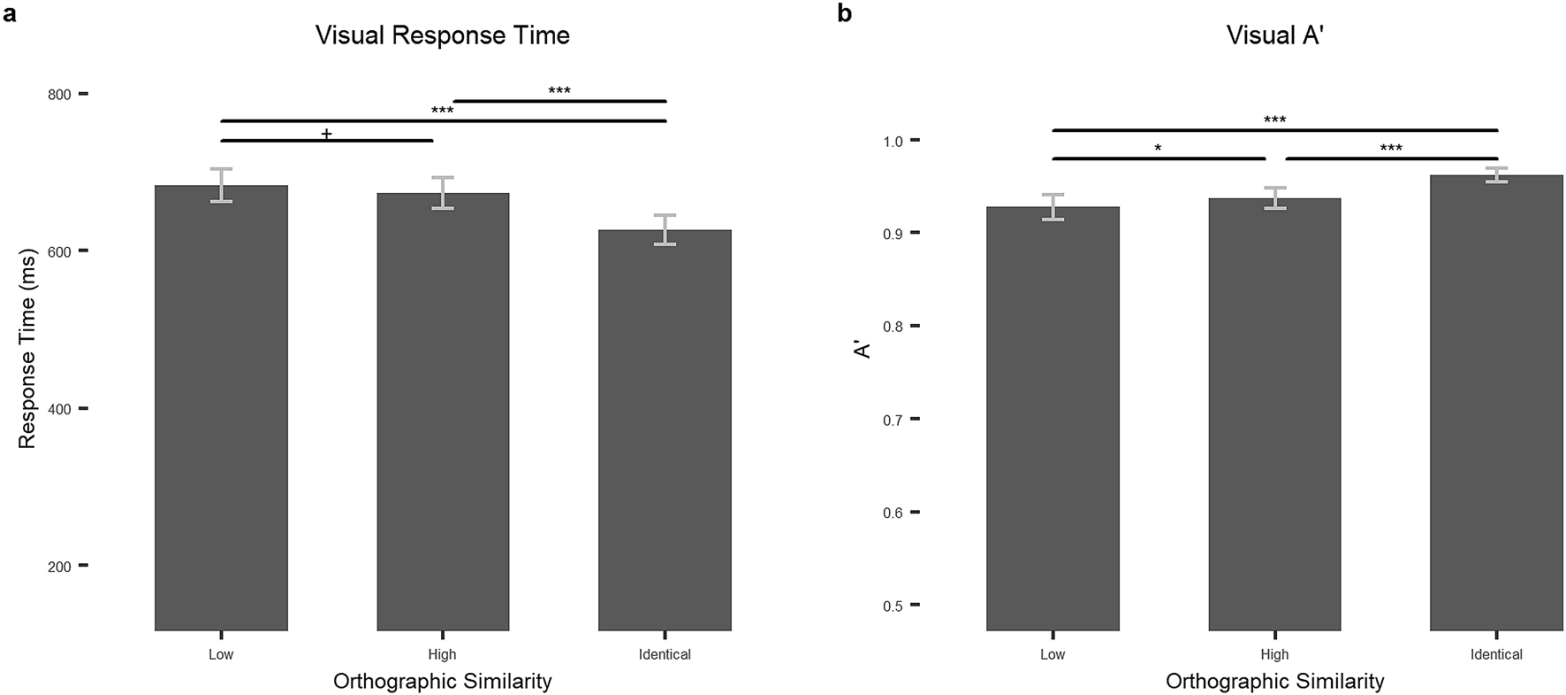
The effects of orthographic similarity on the lexical decision task in the visual modality. (**a**) Response times in miliseconds and (**b**) A’ by orthographic similarity (low, high, and identical) for the visual task. Error bars stand for 95% confidence intervals. N.s.: *p* > .1; + : *p* < .1; *: *p* < .05; **: *p* < .01, ***: *p* < .001.

There was an effect of orthographic similarity on A’, *F*_*1*_(2, 95.304) = 32.325, *p* < 0.001, *η*_*p*_^*2*^ = 0.374, *F*_*2*_(2, 147) = 6.204, *p* = 0.003, *η*_*p*_^*2*^ = 0.078. In the post-hoc tests, we found that identical items had higher signal detection (M = 0.96, SD = 0.03) than both high similarity items (M = 0.94, SD = 0.04), *t*(54) = 6.535, *p* < 0.001, Cohen’s D = 0.881, and low similarity items(M = 0.93, SD = 05) , *t*(54) = 6.699, *p* < 0.001, Cohen’s D = 0.903. High similarity items also had higher signal detection than low similarity items, *t*(54) = 2.274, *p* = 0.081, Cohen’s D = 0.307—see Table 2 and Fig. 5 for by-subject statistics.

We found an effect of orthographic similarity on response time, *F*_*1*_(2, 108) = 75.689, *p* < 0.001, *η*_*p*_^*2*^ = 0.584, *F*_*2*_(2, 147) = 25.164, *p* < 0.001, *η*_*p*_^*2*^ = 0.255. In the post-hoc tests, we found identical items were recognized faster (M = 626.78, SD = 70.26) than both high (M = 673.83, SD = 74.23), *t*(54) = 10.430, *p* < 0.001, Cohen’s D = 1.406, and low similarity items (M = 683.38, SD = 77.88), *t*(54) = 10.426, *p* < 0.001, Cohen’s D = 1.406, but high and low similarity items did not differ, *t*(54) = 1.994, *p* = 0.154, Cohen’s D = 0.269—see Table 2 and Fig. 5 for by-subject statistics.

## Discussion

The objectives of the present study were three-fold: to assess phonological similarity effects in the auditory modality, to assess phonological and orthographic similarity effects and their interactions across modalities, and to evaluate whether orthographically identical words are a special case within the spectrum of orthographic similarity. We had participants do two lexical decision tasks—one in the visual and another in the auditory modality—in their L2, in counterbalanced order.

We found that phonological similarity induced facilitation in the auditory modality, as orthographic similarity did in the visual modality. We also found that the effects of orthographic and phonological similarity produced inhibitory effects across modalities—i.e., phonological similarity causing inhibition in the visual modality and orthographic similarity doing so in the auditory modality. It should be noted that these effects may be qualified by a triple interaction found in the LMEs (provided in the supplementary materials to provide a fuller picture of the data, controlling simultaneously for by participant and by item random effects). In these analyses, we found an interaction between modality, orthographic similarity, and phonological similarity. This interaction suggested that, in the visual modality, phonological similarity worsened performance (lower accuracy and slower response times), but orthographic similarity had no effect, while in the auditory modality there was an interaction such that orthographic similarity worsened performance (reduced accuracy and increased response times) in the low phonological similarity condition, but had no effect on the high similarity condition. Taking into account both analyses, it is possible that the effects of orthography and phonology are not exactly symmetrical between modalities. Finally, we found that orthographically identical words do in fact have a special status, showing disproportionately larger effects in both modalities—facilitatory, in the case of the visual modality, and inhibitory, in the case of the auditory modality.

In the visual modality, we found an orthographic similarity facilitation effect for A’ but not for response times. This result is partially in line with prior studies that describe a facilitatory cognate effect^2–9,40^, which has been found in different domains. For example, some found it in response time only^2,8,9^, others in accuracy only^7^, and finally others in both response time and accuracy^3,5,6^. Importantly, we found that phonological similarity had a somewhat paradoxical effect in which higher phonological similarity hindered performance in the visual modality (both in response time and marginally in A’). These results have been seen before in a study from Dijkstra et al.^5^ in which they found that phonological similarity hindered performance in the visual modality. Our results go beyond this and show the same inhibitory effects for orthographic similarity in the auditory modality (see below for further discussion on orthographic similarity effects in the auditory modality).

It should be noted that not all of the effects that were significant in both the by-participant analyses were significant by-items. This is probably influenced by the fact that we were underpowered in the by-item analyses (which were between items and had only 50 items per category). Nevertheless, our linear mixed effects model results that controlled for by item random effects did align with the by-participant analyses.

Our results provide further evidence of the cross-modal nature of language, while also suggesting that the influence of the L1 during L2 processing is not limited to the modality relevant to the task. First, we show that phonological similarity influences visual word recognition, and vice versa (see below), showing that the non-target language influences target language processing not only within but also across modalities. Furthermore, although intuitively one might think that phonological similarity should improve performance regardless of modality, looking at the particularities of the languages we tested, this is not as straight forward. In the case of an opaque language like English, it makes sense that a cross-modal influence of a transparent language with different phonology, such as Spanish, would hinder performance. In these two languages, the phonological to orthographic correspondences differ greatly, which would naturally bring confusion and spread activation towards other words than the target one, rather than helping the participant hone in on the correct answer.

In the auditory modality, we found that phonological similarity affected signal detection (A’), but not response times, with high similarity items being recognized better than low similarity ones. This aligns with the notion that similarity between L1 and L2 aids word processing, but regarding the auditory modality, this had only been shown so far in production tasks^8,19^. Our results in the auditory modality align with those of Sadat and colleagues^19^ who found that phonological similarity facilitated word production in bilinguals. Our study extends these results to perception and crosses the effects with those of orthographic similarity. We found that orthographic similarity hindered processing in the auditory modality, with high similarity items being recognized slower and less accurately than low similarity ones—although, again, in accuracy we found a possible interaction between types of similarity in the auditory condition. These results are analogous to those mentioned before of Dijkstra et al.^5^ who showed that phonological similarity hindered visual processing. Although a similar inhibitory effect has been found for production in low proficiency participants^18^, this effect had not been described in perception nor in highly proficient bilinguals. This is also in contrast with the traditional cognate effect, where orthographically similar items have a processing advantage over dissimilar ones. Perhaps this contrast with the literature reflects a conflated definition of similarity incorporating both phonological and orthographic similarity. More specifically, using stimuli that align in both areas might suggest a facilitative effect of orthography in the auditory modality that in fact might have been driven by phonology. In more general terms, our results point to a separation between orthographic and phonological representations of words which are relied on differentially according to the task schema. Supporting previous research, similarity between languages aids processing within the same modality and shows that both languages are active even in unilingual tasks^10^.

Orthographically identical words showed effects above and beyond those of high similarity words. In other words, although the increase in similarity between high orthographic similarity and identical items was often minimal—e.g., ‘band’ (Spanish: ‘banda’) is not identical but ‘taxi’ (Spanish: ‘taxi’) is—the effect of similarity was disproportionately larger for orthographically identical words. In the case of facilitation in the visual modality (both for A’ and response time), this result is in line with previous findings that showed a larger facilitation effect for orthographically identical words over high similarity items^8,10,13^. The current study extends these findings to the auditory modality, in which we found a disproportionate level of inhibition by orthographically identical words, slowing response times compared to high similarity items, which did not differ from low similarity items. Importantly, this suggests a nonlinear relationship that places identical items in a special category. On the other hand, our orthographic and phonological results contrast with those of Schwartz and colleagues^8^ who found an interaction between orthographic and phonological similarity. But, importantly, they mixed identical and high orthographic similarity items and found that very high similarity words (including identical items) were produced faster than high orthographic similarity items, but that phonological dissimilarity hindered performance. Although Schwartz and colleagues observed facilitation in this production task, others have found inhibition for cognates in production, depending on task difficulty^41^. Therefore, this contrast with our results might be due to differences in the task (production versus perception) or it might relate to task difficulty. This would be in line with previous monolingual research showing both facilitation and inhibitory effects of semantic similarity^42,43^, phonological similarity^44^, and phonological neighborhood density^45^ depending on various factors. This difference also suggests that perhaps orthographically identical words, due to their “special status”, are affected by phonological similarity in a different way as compared to items that have highly orthographic similarity across languages.

It is also important to point out that there are several aspects of our study that are unique and may have influenced the effects that we found. The first is that the target language was opaque, but the native language of participants was transparent. As a participant heard the string, he/she built a predicted orthographic representation based on the sublexical phonological units (the “transcription”), which had to be compared to the lexical representation. In transparent languages, such as Spanish or Italian, this is quite a straightforward process, with mostly one-to-one correspondences between phonemes and graphemes. On the other hand, in English, this process is more difficult, particularly for participants with a transparent L1, who are used to relying mostly on one-to-one phoneme-to-grapheme relationships. If a transparent target language was used as the target language in a similar experiment, one of two things might happen: if the grapheme/phoneme correspondences align between languages, this should lead to facilitatory rather than inhibitory effects across modalities; if they do not align, this might still lead to confusion and delays in processing. Another interesting follow-up would be to test participants with an opaque L1. They might not be bothered by misalignment at all and might show no effect of orthographic similarity in the auditory modality and vice versa.

Another issue relates to accent, as the aural items were recorded with a native accent, which participants were not used to hearing (Spanish natives being more used to listening to Spanish accented English than English produced by native speakers). The accent used in the stimuli of the present study being a native accent, what they heard probably did not match what they are used to hearing nor how they would pronounce this word. Native Spanish speakers probably had representations of English words built through listening to a majority of non-native speakers from their country and influenced by the phonology of their L1. It is not clear whether the results would be the same if the stimuli had been produced with a foreign accent matching their L1. Future studies should test the relative influence of this factor.

To our knowledge, this is the first experiment to orthogonally manipulate orthographic and phonological similarity across modalities, to test the effects of interlingual similarity on perception in the auditory modality, and to show that orthographic similarity hinders auditory performance and vice versa. Our results fall in line with the co-activation account frequently mentioned in the literature, showing effects of the unattended language, in both modalities with both measures of interlingual similarity (orthographic and phonological). The main question that remains is why there is facilitation within modality—phonological in the auditory modality and orthographic in the visual—but inhibition across modalities—orthographic in the auditory and phonological in the visual.

According to the BIA and BIA + models, language co-activation aftereffects occur when orthography, phonology, and semantic features are similar across languages^10^. These models suggest that both languages of a bilingual are co-activated in parallel when they are performing a unilingual task. As a consequence, any similarity between words in both languages should benefit word recognition since language co-activation should help this process, which does not completely align with our findings. It is worth noting that these models do not address differences between modalities and thus seem to assume equal effects. In our experiment, we found that the effect of similarity was contingent upon the modality of presentation and whether the type of similarity aligned with the task—i.e., orthographic with visual and phonological with auditory. On a theoretical level, our results suggest that models of bilingual processing—such as the BIA + model—need to contemplate processing in the auditory modality as well as making a distinction between orthography and phonology. In other words, they need to account for modality and cross-modality interactions: Models should account for the fact that representations in the other language are not only activated in the modality of the task, but also in the irrelevant modality, and that facilitatory/inhibitory interactions happen across modalities. At the same time, the transparent or opaque nature of each language might also be relevant for understanding the influence of one on the other: in order to understand bilingual word processing, we need to incorporate the characteristics of the languages—both independently and in relation to each other—to our models.

Furthermore, a similar inhibitory effect has been attributed to increased competition between words in the target and non-target languages^18^, which would explain our results as well, particularly in the case of orthographically identical words with different phonology. This makes the relationship between the languages and the characteristics of each language relevant factors when studying their co-activation. More specifically, orthographic and phonological similarity effects may vary according to whether the languages share grapheme to phoneme correspondences or whether they are similar phonetically. As we mentioned before, given that other studies have not manipulated phonological and orthographic similarity orthogonally, it is difficult to compare results as, in previous studies, the effects of orthography might have been masked by those of phonology, or vice versa.

With respect to methodology, this study shows that we need to quantify and control for word similarity in the seemingly irrelevant modality when testing bilinguals. The inhibitory effects across modalities highlight the importance of taking into account how the phonological and orthographic codes of each of the languages of a bilingual relate when testing them. It is not enough to simply say that a word is “similar in form” to its translational equivalent, as cognates are often defined. It is necessary to qualify which aspects of form—be it phonological or orthographic—are similar as well as whether this aligns with the presentation modality.

In conclusion, the results of this study suggest an interference between lexical item representations in different modalities for bilinguals. Furthermore, this study paves the way for further research on the dissociation between orthographic and phonological representations, and the respective effects of orthographic and phonological similarity within and across modalities. Understanding how the different representations of a word interact between languages is essential for our understanding of how the bilingual mind accommodates two full languages with their respective orthographic and phonological codes.

## Supplementary Information

Supplementary Information.

## Data Availability

All data, scripts, and stimuli are available at https://osf.io/mqsbj/?view_only=a6f2f4e617874595bd429914f1a0f57c
