# Supplementary material for: Inhibitory and facilitatory effects of phonological and orthographic similarity on L2 word recognition across modalities in bilinguals
Source: Sci Rep. 2021 Jun 17;11:12812. doi: 10.1038/s41598-021-92259-z (PMC8211678; doi:10.1038/s41598-021-92259-z)
Supplement: Supplementary file 1 — Supplementary Information. [file 41598_2021_92259_MOESM1_ESM.pdf]

Inhibitory and facilitatory effects of phonological and orthographic similarity on L2 word recognition across modalities in bilinguals

Candice Frances[1,2*], Eugenia Navarra-Barindelli[1,2], and Clara D. Martin[1,3]

[1] BCBL, Basque Center on Brain, Language and Cognition; Donostia, Spain

[2] Department of Social Sciences and Law, UPV/EHU, Donostia, Spain

[3] Ikerbasque, Basque Foundation for Science, Bilbao, Spain

*c.frances@bcbl.eu, candice.frances@ncf.edu

LME results

The following are the linear mixed effect model analyses. In all cases, analyses were run using linear mixed-effects models in R, lme4[1] and lmerTest package [2]. Two-level categorical predictors (modality, phonological similarity, orthographic similarity, session number, and order—visual or auditory first) were coded as -0.5 and 0.5 (see table below). Subject and item intercepts were included as random effects.

Table of factor coding

	-.5	.5
Orthographic similarity	Cognate	Non Cognate
Phonological similarity	Cognate	Non Cognate
Word condition	Pseudoword	Real Word
Location	Madrid	Murcia
Modality	Auditory	Visual
Order	Auditory First	Visual First

The models included all fixed effects of interest, both those we manipulated—modality, phonological similarity, and orthographic similarity—and those we simply controlled for—word frequency, English level of participants, order of presentation, trial number, and session number. We included random intercepts for participants and items.

Accuracy

The first model had accuracy (0 incorrect; 1 correct) as the dependent variable and modality (visual or auditory), phonological similarity (cognate or noncognate), and orthographic similarity (cognate or noncognate) as well as their interactions as fixed effects. We also included the logarithmic transformation of word frequency (as reported in CLEARPOND[3]), English level of participants (as measured by their performance on the English LexTALE[4]), order of presentation (visual session first or auditory session first), trial number rescaled (trial number from 1 to 600 rescaled to from 0 to 1), and session number (first or second). Given that we had no specific and to avoid overfitting, we excluded their interactions from the model. In addition, we included the random intercepts for subject and item. This model converged and the results were interpretable.

In the model, there was a main effect of frequency (β = 2.667, *SE* = .529, z = 6.932, *p* < .001) in the expected direction of higher accuracy for higher frequency words, of English level (β = 4.375, *SE* = 1.195, z = 3.661, *p* < .001) in the expected direction of higher accuracy in participants with higher scores in the LexTALE, of trial (β = -.527, *SE* = .120, z = -4.402, *p* < .001) such that accuracy decreased in later trials, and of session (β = -.178, *SE* = .071, z = -2.510, *p* =.012) such that participants did better in the first than the second session. There was no effect of session order (β = .034, *SE* = .228, z = .151, *p* = .880).

With respect to the variables of interest, there was a significant main effect of modality (β = .642, *SE* = .072, z = 8.892, *p* < .001), such that participants had higher accuracy in the visual modality. There was a marginal main effect of phonological similarity (β = .365, *SE* = .187, z = 1.950, *p* = .051), such that phonological non-cognates led to higher accuracy. There was no main effect of orthographic similarity (β = .045, *SE* = .187, z = .242, *p* = .808). These effects were qualified by an interaction between modality and phonological similarity (β = .445, *SE* = .143, z = 3.112, *p* = .002) such that phonological similarity lead to lower accuracy in the visual modality, but not the auditory modality as well as an interaction between modality and orthographic similarity (β = -.337, *SE* = .143, z = -

2.359, p = .018) such that orthographic similarity lead to lower accuracy in the auditory modality, but not the visual modality. There was no interaction between orthographic and phonological similarity overall (β = .358, SE = .373, z = .958, p = .338). Finally, there was a triple interaction between modality, phonological, and orthographic similarity (β = -.807, SE = .286, z = -2.82, p = .005), such that in the visual modality phonological similarity reduced accuracy, but orthographic similarity had no effect, and in the auditory modality there was an interaction such that orthographic similarity decreased accuracy in the low phonological similarity condition, but had no effect on the high similarity condition, leading to an overall decrease in performance with high orthographic similarity.

Response time

We then ran the same analyses on response time as with accuracy. The model had response time in miliseconds as the dependent variable and modality (visual or auditory), phonological similarity (cognate or noncognate), and orthographic similarity (cognate or noncognate) as well as their interactions as fixed effects. We also included the logarithmic transformation of word frequency (as reported in CLEARPOND[3]), English level of participants (as measured by their performance on the English LexTALE[4]), order of presentation (visual session first or auditory session first), trial number rescaled (trial number from 1 to 600 rescaled to from 0 to 1), and session number (first or second). Given that we had no specific and to avoid overfitting, we excluded their interactions from the model. In addition, we included the random intercepts for subject and item. This model converged and the results were interpretable.

In the model, there was a main effect of frequency [β = -128.643, SE = 23.214, $t(199)$ = -5.543, p < .001] in the expected direction of faster response times for higher frequency words, of trial [β = 251.443, SE = 2.956, $t(11305.868)$ = 85.075, p < .001] such that accuracy decreased in later trials. There was no effect of session order [β = -11.965, SE = 26.365, $t(30)$ = -.454, p = .653], of English level [β = -121.318, SE = 138.922, $t(30)$ = -.873, p = .389], nor of session number [β = -1.265, SE = 2.945, $t(11275)$ = -.430, p = .667.

With respect to the variables of interest, there was a significant main effect of modality [β = 251.443, SE = 2.956, $t(11306)$ = 85.075, p < .001], such that participants were slower in the visual modality. There was no main effect of phonological similarity [β = -4.641, SE = 8.000, $t(191)$ = -.580, p = .562] and no main effect of orthographic similarity [β = -8.909, SE = 7.996, $t(191)$ = -1.114, p = .267]. Main effects were qualified by an interaction between modality and phonological similarity [β =-12.318, SE = 5.906, $t(11305)$ = -2.086, p = .037] such that phonological similarity lead to slower response times in the visual modality, but not the auditory modality as well as an interaction between modality and orthographic similarity [β = 17.339, SE = 5.906, $t(11305)$ = 2.936, p = .003] such that orthographic similarity lead to slower response times in the auditory modality, but not the visual modality. There was no interaction between orthographic and phonological similarity overall [β = -18.426, SE = 15.952, $t(191)$ = -1.155, p = .249]. Finally, there was a marginally significant triple interaction between modality, phonological, and orthographic similarity [β = 20.336, SE = 11.816, $t(11305)$ = 1.722, p = .085], such that in the visual modality phonological similarity increased response times, but orthographic similarity had no effect and in the auditory modality there was an interaction such that orthographic similarity increased response times in the low phonological similarity condition, but had no effect on the high similarity condition, leading to an overall increase in response times with high orthographic similarity.

References

1. Bates, D., Maechler, M., Bolker, B. & Walker, S. Fitting linear mixed-effects models using lme4. *J. Stat. Softw.* **67**, 1–48 (2015).

2. Kuznetsova, A., Brockhoff, P. B. & Christensen, R. H. B. lmerTest package: Tests in linear mixed effects models. *J. Stat. Softw.* **82**, 1–26 (2017).

3. Marian, V., Bartolotti, J., Chabal, S. & Shook, A. Clearpond: Cross-linguistic easy-access resource for phonological and orthographic neighborhood densities. *PLoS One* **7**, (2012).

4. Lemhöfer, K. & Broersma, M. Introducing LexTALE: A quick and valid lexical test for advanced learners of English. *Behav. Res. Methods* **44**, 325–343 (2012).
